# High dietary folate in pregnant mice leads to pseudo-MTHFR deficiency and altered methyl metabolism, with embryonic growth delay and short-term memory impairment in offspring

**DOI:** 10.1093/hmg/ddx004

**Published:** 2017-01-06

**Authors:** Renata H. Bahous, Nafisa M. Jadavji, Liyuan Deng, Marta Cosín-Tomás, Jessica Lu, Olga Malysheva, Kit-Yi Leung, Ming-Kai Ho, Mercè Pallàs, Perla Kaliman, Nicholas D.E. Greene, Barry J. Bedell, Marie A. Caudill, Rima Rozen

**Affiliations:** 1 Departments of Human Genetics and Pediatrics, Research Institute of the McGill University Health Center, Montreal, Quebec, Canada; 2 Department of Neuroscience, Carleton University, Ottawa, Ontario, Canada; 3 Pharmacology Unit, Faculty of Pharmacy, Institut de Neurociència Universitat de Barcelona (IBUB), Nucli Universitari de Pedralbes, Barcelona, Spain; 4 Division of Nutritional Sciences and Genomics, Cornell University, Ithaca, NY, USA; 5 Developmental Biology and Cancer Programme, Great Ormond Street Institute of Child Health, University College London, London, United Kingdom; 6 Department of Neurology and Neurosurgery, McGill University, Montreal, Quebec, Canada; 7 Institute of Biomedical Investigation of Barcelona, Spanish National Research Council, Barcelona, Spain; 8 Center for Mind and Brain, University of California Davis, Davis, CA, USA

## Abstract

Methylenetetrahydrofolate reductase (MTHFR) generates methyltetrahydrofolate for methylation reactions. Severe MTHFR deficiency results in homocystinuria and neurologic impairment. Mild MTHFR deficiency (677C > T polymorphism) increases risk for complex traits, including neuropsychiatric disorders. Although low dietary folate impacts brain development, recent concerns have focused on high folate intake following food fortification and increased vitamin use. Our goal was to determine whether high dietary folate during pregnancy affects brain development in murine offspring. Female mice were placed on control diet (CD) or folic acid-supplemented diet (FASD) throughout mating, pregnancy and lactation. Three-week-old male pups were evaluated for motor and cognitive function. Tissues from E17.5 embryos, pups and dams were collected for choline/methyl metabolite measurements, immunoblotting or gene expression of relevant enzymes. Brains were examined for morphology of hippocampus and cortex. Pups of FASD mothers displayed short-term memory impairment, decreased hippocampal size and decreased thickness of the dentate gyrus. MTHFR protein levels were reduced in FASD pup livers, with lower concentrations of phosphocholine and glycerophosphocholine in liver and hippocampus, respectively. FASD pup brains showed evidence of altered acetylcholine availability and *Dnmt3a* mRNA was reduced in cortex and hippocampus. E17.5 embryos and placentas from FASD dams were smaller. MTHFR protein and mRNA were reduced in embryonic liver, with lower concentrations of choline, betaine and phosphocholine. Embryonic brain displayed altered development of cortical layers. In summary, high folate intake during pregnancy leads to pseudo-MTHFR deficiency, disturbed choline/methyl metabolism, embryonic growth delay and memory impairment in offspring. These findings highlight the unintended negative consequences of supplemental folic acid.

## Introduction

Folate derivatives are critical for brain development and neurotransmitter synthesis because they provide one-carbon units for nucleotide synthesis and methylation reactions (1). One of the most studied enzymes in the folate metabolic pathway is methylenetetrahydrofolate reductase (MTHFR, EC 1.5.1.20), a ubiquitous enzyme which generates 5-methyltetrahydrofolate (5-methylTHF) for remethylation of homocysteine to methionine (2). Methionine is the precursor of S-adenosylmethionine (SAM), the primary methyl donor in most mammalian methylation reactions. An alternate folate-independent pathway for methionine synthesis is present primarily in liver and kidney; it uses the choline metabolite betaine as a methyl donor. Disturbances in folate metabolism can be genetic and/or dietary and often result in compensatory disturbances in choline metabolism. Choline is required to synthesize the neurotransmitter acetylcholine and critical phospholipids for membrane integrity (3,4).

Relatively rare severe deficiencies in MTHFR (usually <20% of enzyme activity) are a cause of homocystinuria, an inborn error of metabolism characterized by a variety of neurologic symptoms including developmental delays, motor disturbances and brain atrophy (5). A mild deficiency in MTHFR, due to the 677C > T (A222V) polymorphism, is present in the homozygous state in 10–15% of many Caucasian populations and encodes a thermolabile enzyme with ∼30% residual activity (2). This SNP has been reported to increase risk for neural tube defects, pregnancy complications and neuropsychiatric diseases such as schizophrenia and autism (3,6–8). To study these complex traits, we developed mouse models that mimic these MTHFR deficiencies (9). *Mthfr^-/-^* mice, a model for homocystinuria, have short-term memory impairment, altered hippocampal morphology and disturbed acetylcholine metabolism (10). Heterozygous MTHFR deficiency in dams (*Mthfr^+/-^* mice) resulted in memory impairment in their pups (11).

Folate cannot be synthesized by humans and must be acquired from the diet (12). Low maternal folate intake during pregnancy is a risk factor for neural tube defects and other birth defects, as well as other pregnancy complications including intrauterine growth restriction (3). Low maternal folate intake has also been associated with disturbances in offspring brain function, since the developing brain is sensitive to nutrient depletion during gestation and early development. In both humans and rodents, folate deficiency during pregnancy has been associated with cognitive impairment, increased anxiety and depression (13,14). In mice, maternal folate-deficient or choline-deficient diets lead to increased apoptosis in fetal brain, particularly the hippocampus (11,15).

In a public health effort to reduce the incidence of neural tube defects, grains have been fortified with folic acid in over 50 countries. The recommended daily intake for adults is 0.4 mg with a recommended tolerable upper intake level of 1 mg (16). However, there are groups in the population that are consuming 4–5 mg/day, primarily due to the increased use of vitamin supplements (17,18). Concerns have emerged about the possible consequences of high folic acid intake; these concerns include increased growth of sub-clinical tumors, the possibility of masking B_12_ deficiency and impaired immune function (16,19). We have been investigating potential adverse outcomes of high folic acid intake in mice. We found that adult male mice fed a folic acid supplemented diet (FASD, 10× the recommended amount for rodents) for 6 months developed a pseudo-MTHFR deficiency, with hepatic injury (20).

The use of vitamin supplements is particularly important for women of childbearing age, although high-dose supplementation with folic acid raises questions about possible negative effects on their offspring, particularly on brain development (21). Therefore, in this study we aimed to determine whether high maternal folic acid intake in mice affected brain function or metabolism in their pups and embryos. Towards this end, we fed wild-type female mice a control diet (CD) or FASD for 5 weeks prior to mating, and maintained the diets throughout pregnancy and lactation, to assess brain development in offspring. We found that offspring of mothers fed FASD had intra-uterine growth delay, short-term memory impairment and altered brain development. Maternal and offspring pseudo-MTHFR deficiency with disturbances in choline/methyl metabolism are likely to have contributed to these outcomes.

## Results

### Three-week-old pups born to FASD mothers had short-term memory impairment and reduced hippocampal area

**Figure 1:**
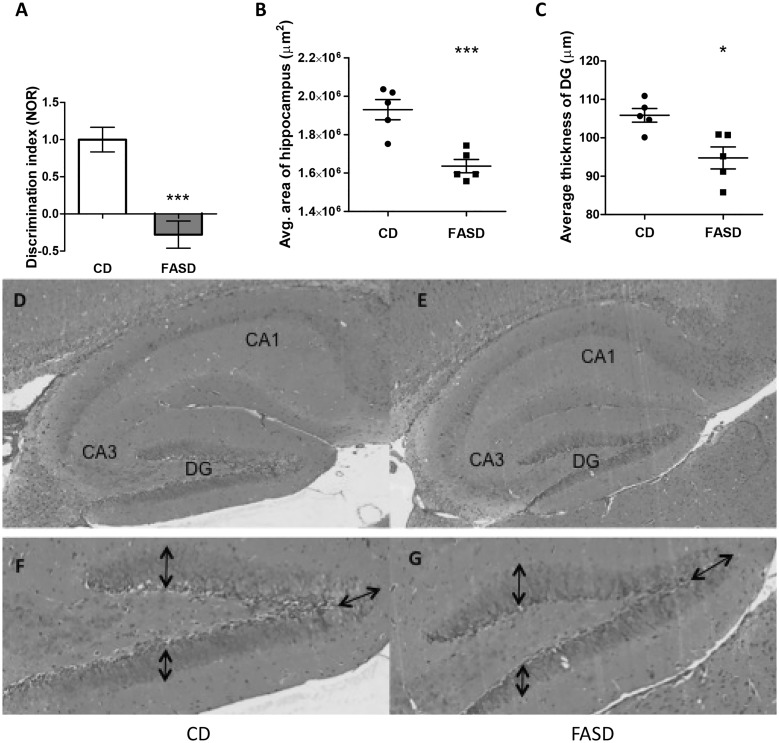
Pups born to mothers fed high folate diet had short-term memory impairment and reduced hippocampal area. (**A**) Three**-**week-old male mice were tested using the NOR. The discrimination index is the ratio of time spent with the novel object versus time with the familiar object; a negative discrimination index indicates short-term memory impairment. Data from two independent experiments are shown (*n* = 15–20/group). (**B**) The average area of the hippocampus and (**C**) the average thickness of the dentate gyrus (DG) were reduced in FASD pups (*n* = 5/group). Representative images of hippocampus and DG from CD pup (**D** and **F**, respectively) and FASD pup (**E** and **G**, respectively) are shown. Arrows in the DG indicate where the thickness measurements were performed. Values are means ± SEM; *P < 0.05, ***P < 0.005.

There were no differences in body weights of pups between the two dietary groups (Supplementary Material, Fig. S1A). Behavioral testing was performed on 3-week-old male pups to assess brain function. In the ladder beam test for measurement of gait, there were no differences in percent error or movement score (Supplementary Material, Fig. S1B). In the open field test, which evaluates anxiety, there were no differences in the activity pattern or number of field entries (Supplementary Material, Fig. 1C). To assess memory, the novel object recognition test (NOR) and the Y-maze test were performed. No differences were observed in the Y-maze test suggesting there were no differences in spatial memory (Supplementary Material, Fig. 1D). However, mice from FASD mothers had a negative discrimination index in the NOR, i.e. these pups spent significantly less time with the novel object than with the familiar object (Fig. 1A;*P* < 0.005, unpaired *t*-test). These findings suggest that pups from FASD mothers have short-term memory impairment, a phenotype that was also observed in *Mthfr*^*-/-*^ mice (10). Since we had previously observed that *Mthfr*^*-/-*^ mice had reduced hippocampal volume, we measured the area of the hippocampus and the thickness of the dentate gyrus (DG) in sagittal brain sections of pups; both were reduced due to FASD (*P* < 0.005 and *P* < 0.05, respectively, unpaired *t*-test) (Fig. 1B–G).

### Maternal FASD during pregnancy and lactation led to pseudo-MTHFR deficiency in offspring liver

**Figure 2:**
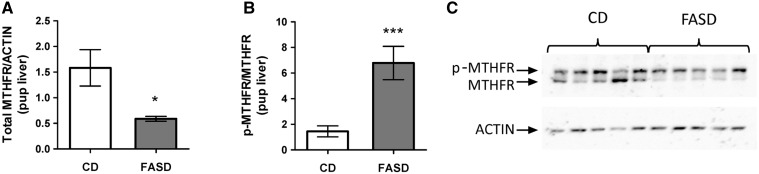
Pups born to mothers fed high folate diet had pseudo-MTHFR deficiency in liver. (**A**) Total immunoreactive MTHFR protein was reduced in livers of FASD pups. (**B**) The ratio of the phosphorylated isoform (p-MTHFR, less active) to the non-phosphorylated isoform (MTHFR) was increased in FASD pups. (**C**) Representative Western blot of MTHFR in offspring liver. Values are means ± SEM from 5 mice/group; *P<0.05, ***P<0.005.

We previously reported that FASD leads to pseudo-MTHFR deficiency in adult male liver (20). We therefore measured MTHFR protein levels in liver of 3-week-old offspring. We found a 3-fold decrease in total MTHFR protein (Fig. 2A and C;*P* < 0.05, unpaired *t*-test) and a 4-fold increase in the ratio of the phosphorylated to non-phosphorylated isoform of MTHFR (Fig. 2B and C;*P* < 0.005, unpaired *t*-test). Decreased phosphorylation has been reported to increase MTHFR activity by 1.2 fold *in vitro*; the effect of phosphorylation *in vivo* has not been examined (22). No changes were detected at the mRNA level (data not shown). These data are consistent with our previous report and suggest that offspring born to FASD mothers have pseudo-MTHFR deficiency.

We also measured MTHFR levels in the cortex and hippocampus, two brain regions that impact performance in the NOR test, but no differences in total immunoreactive MTHFR levels were detected (data not shown). Consistent with this observation, there were no differences in the percentages of THF and its one-carbon substituted derivatives in the hippocampus between the two dietary groups (Supplementary Material, Table S1).

### Choline metabolism was disturbed in liver and brain of FASD pups

**Figure 3:**
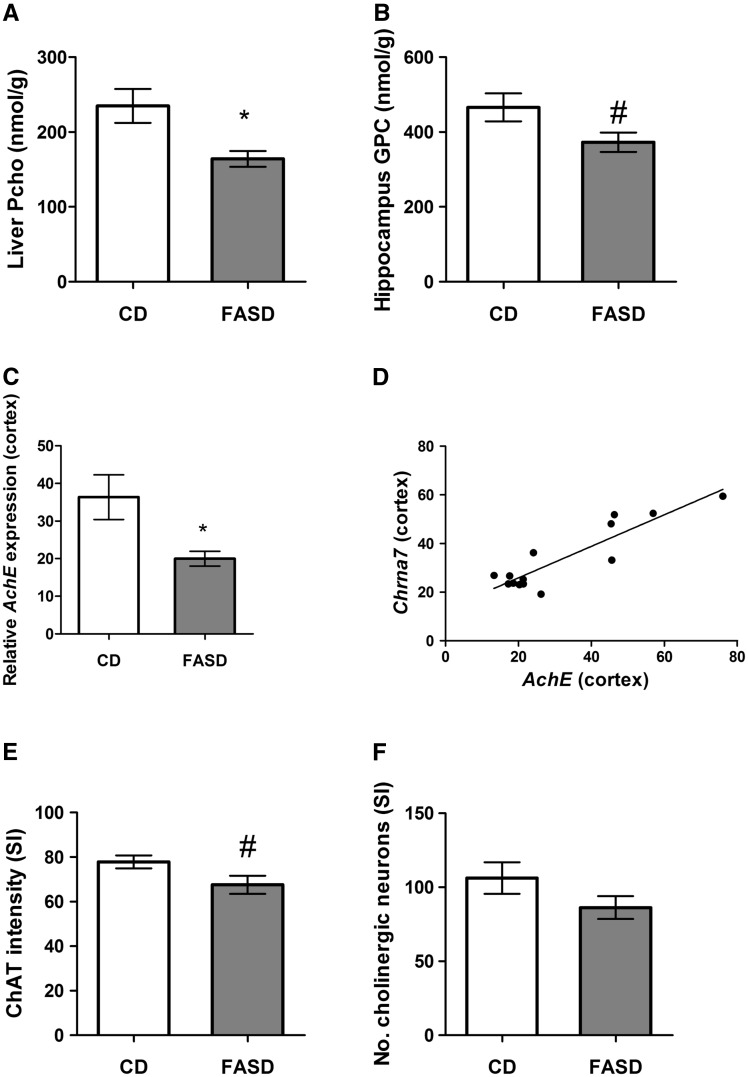
Pups born to mothers fed high folate diet had disturbances in choline and acetylcholine metabolism. (**A**) The concentration of phosphocholine (PCho), the storage form of choline, was significantly decreased in liver (*n* = 6/group). (**B**) In hippocampus, there was a trend towards reduced glycerophosphocholine (GPC) in FASD pups (*n* = 7/group, #*P* = 0.06). (**C**) mRNA expression of acetylcholinesterase (*AchE)* was reduced in cortex of FASD pups (*n* = 7/group). (**D**) mRNA levels of the nicotinic receptor of acetylcholine, *Chrna7* and *AchE* in cortex were significantly positively correlated (*n* = 7/group; *r* = 0.894, *P* < 0.05). ChAT staining to measure (**E**) staining intensity and (**F**) numbers of cholinergic neurons in the Substantia Innominata (SI). There was a trend towards reduced intensity of ChAT staining in the SI of FASD pups (*n* = 5/group, #*P* = 0.07) but no significant difference in number of cholinergic neurons. Values are means ± SEM; *P < 0.05.

In several other studies, we have shown that disturbances in folate metabolism are often accompanied by disturbances in choline metabolism, because these two pathways intersect in the provision of methyl donors for methionine/SAM synthesis (10,20,23). We therefore measured SAM, SAH, choline and related metabolites in offspring liver, the primary organ of one-carbon metabolism. We did not observe differences in SAM, SAH, choline, betaine, glycerophosphocholine (GPC), phosphatidylcholine, sphingomyelin or lysophosphatidylcholine (Supplementary Material, Table S2). However, there was a significant 30% decrease in phosphocholine (PCho), the primary storage form of choline (Fig. 3A;*P* < 0.05, unpaired *t*-test).

We also measured choline metabolites in hippocampus (Supplementary Material, Table S2) and observed a trend for decreased levels of GPC in offspring (Fig. 3B;*P* = 0.06, unpaired *t*-test), suggesting an increased conversion of GPC to choline to replenish choline pools.

Choline is a precursor of acetylcholine (Ach), a neurotransmitter that plays a role in memory (4). mRNA levels of acetylcholinesterase (*AchE)*, which cleaves acetylcholine at the synaptic cleft, were measured in cortex and hippocampus. *AchE* mRNA was significantly reduced in the FASD group in cortex (Fig. 3C;*P* < 0.05, unpaired *t*-test with Welsh’s correction). Cortical mRNA levels of the nicotinic receptor of acetylcholine, *Chrna7*, were decreased in the FASD group but did not reach statistical significance (data not shown). However, we observed a significant correlation between *AchE* and *Chrna7* levels in cortex (Fig. 3D;*r* = 0.894, *P* < 0.05). *AchE* and *Chrna7* mRNA levels did not change in hippocampus (data not shown).

We also measured immunoreactive protein levels of choline acetyltransferase (ChAT), the enzyme that synthesizes Ach from choline; there were no differences in cortical or hippocampal extracts (data not shown). However, when we stained for cholinergic neurons in the Substantia Innominata (SI), which project to the cortex, we found a trend for reduced ChAT staining in FASD pups (Fig. 3E;*P* = 0.07, unpaired *t*-test). There was no significant difference in the number of cholinergic neurons (Fig. 3F).

### FASD pups had reduced expression of *Dnmt3a*

**Figure 4:**
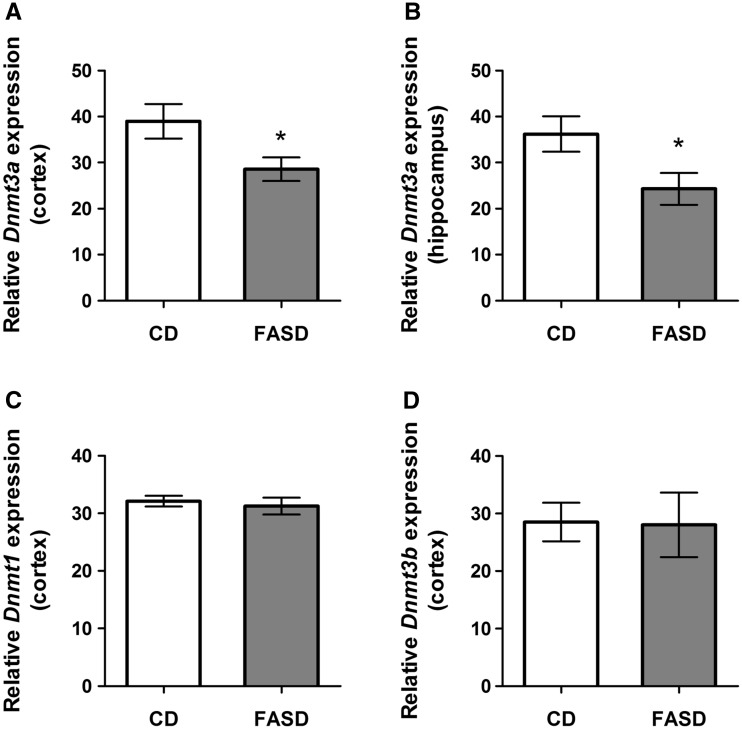
Pups of FASD mothers had reduced expression of *Dnmt3a* mRNA in cortex and hippocampus. There was a significant decrease of *Dnmt3a* mRNA due to FASD in (**A**) cortex (*n* = 7/group) and (**B**) hippocampus of FASD pups (*n* = 5/group). (**C**) *Dnmt1* and (**D**) *Dnmt3b* mRNA were not changed by diet in cortex. Values are means ± SEM; *P < 0.05.

Maternal high folic acid intake can result in methylation changes in the brain of offspring (24), and DNA methyltransferases have been shown to be involved in memory (25). We therefore measured mRNA levels of DNA methyltransferases in offspring cortex and hippocampus. We observed a significant decrease in *Dnmt3a* mRNA in cortex and hippocampus (Fig. 4A and B, respectively; *P* < 0.05, unpaired *t*-test). We did not observe changes in *Dnmt1* (Fig. 4C) or *Dnmt3b* (Fig. 4D) in cortex, suggesting that this effect is specific to the *de novo* methyltransferase *Dnmt3a.*

### FASD resulted in pseudo-MTHFR deficiency and altered folate and choline metabolism in lactating mothers

**Figure 5:**
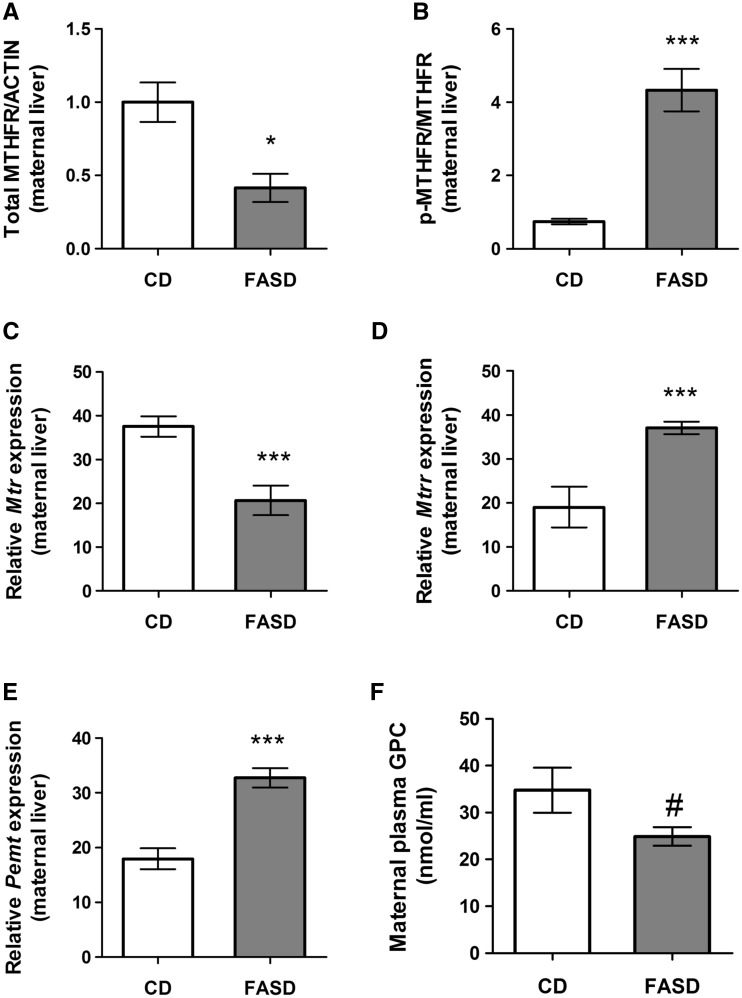
High folic acid intake altered folate metabolism in liver of lactating mothers and led to changes in choline metabolism. (**A**) Total MTHFR protein was reduced in FASD mothers (*n* = 5/group). (**B**) The ratio of the phosphorylated MTHFR isoform (less active) to the non-phosphorylated isoform was increased in the FASD group (*n* = 5/group). mRNA expression of (**C**) methionine synthase (*Mtr*) was reduced, (**D**) methionine synthase reductase (*Mtrr)* was increased and (**E**) *Pemt* was increased due to FASD (*n* = 7/group). (**F**) There was a trend towards lower GPC due to diet in maternal plasma, (*n* = 7/group, #*P* = 0.06). Values are means ± SEM; *P < 0.05, ***P < 0.005.

There were no differences in maternal body weights between groups (CD: 24.8 ± 1.8; FASD: 26.5 ± 1.7). As seen in offspring, there was a decrease in total MTHFR protein in maternal liver (Fig. 5A;*P* < 0.05, unpaired *t*-test) and an increased ratio of phosphorylated to non-phosphorylated MTHFR (Fig. 5B;*P* < 0.005, unpaired *t*-test).

We measured mRNA expression of other important enzymes in one-carbon metabolism. Methionine synthase (*Mtr)* is the enzyme responsible for remethylating homocysteine to methionine using 5-methylTHF generated by MTHFR. In maternal liver, *Mtr* mRNA levels significantly decreased due to diet (Fig. 5C;*P* < 0.005, unpaired *t*-test). mRNA for methionine synthase reductase (*Mtrr)*, the enzyme required to activate *Mtr*, was increased (Fig. 4D;*P* < 0.005, unpaired *t*-test), possibly as a compensatory mechanism for reduced *Mtr.*

Phosphatidylethanolamine methyltransferase (*Pemt)* converts phosphatidylethanolamine into phosphatidylcholine, the precursor of GPC (26). We observed increased *Pemt* mRNA levels due to diet (Fig. 5E;*P* < 0.005, unpaired *t*-test). Choline metabolites were measured in maternal liver and plasma. No changes were observed in liver or plasma for choline, betaine, phosphatidylcholine, PCho, sphingomyelin or lysophosphatidylcholine (Supplementary Material, Table S3). However, there was a trend towards reduced GPC concentrations in maternal plasma (Fig. 5F;*P* = 0.06, unpaired *t*-test). The increase in *Pemt* may be a compensatory mechanism to increase circulating GPC for the generation of choline.

### Embryos and placentas of FASD mothers were smaller at E17.5

**Figure 6:**
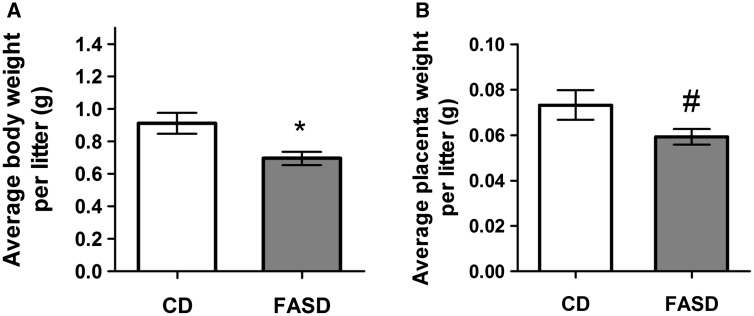
Embryos and placentas from FASD mothers were smaller at E17.5. Weights of embryos and placentas were measured at sacrifice. (**A**) FASD led to lower mean body weights/litter of embryos. (**B**) There was a trend toward lower placental weights/litter (#*P* = 0.08). Values are means ± SEM of 6 l/group; *P < 0.05.

To understand the effects of FASD during early brain development, we collected maternal and embryonic tissues at E17.5 from mothers fed CD and FASD. There were no differences in maternal body weights (CD: 29.9 ± 1.7; FASD: 30.1 ± 2.1) or litter sizes (CD: 6 ± 1.5; FASD: 8.3 ± 0.3) between groups. However, average fetal body weight per litter was significantly reduced in the FASD group (Fig. 6A;*P* < 0.05, unpaired *t*-test) and there was a trend towards reduced placental weight per litter (Fig. 6B;*P* = 0.08, unpaired *t*-test), suggesting that a high folate diet during pregnancy leads to growth delay in embryos.

### FASD embryos showed reduced expression of one-carbon enzymes in liver, reduced hepatic choline metabolites and altered brain development

**Figure 7:**
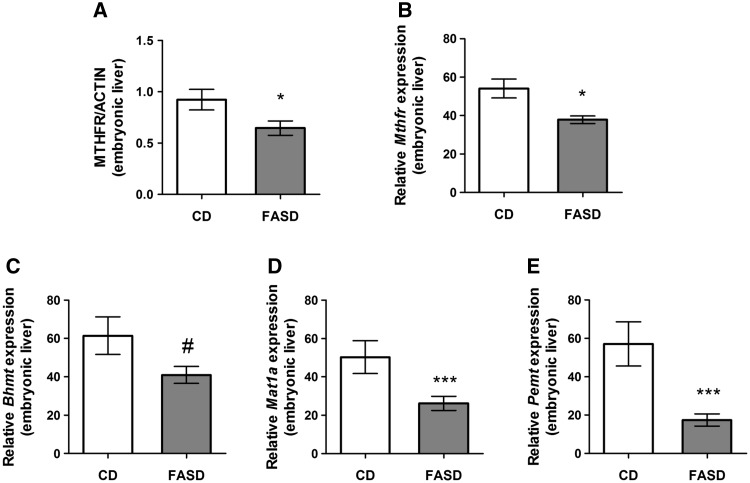
Maternal FASD altered expression of one-carbon enzymes in liver of E17.5 embryos. (**A**) Total MTHFR protein and (**B**) *Mthfr* mRNA was reduced in liver of E17.5 FASD embryos. (**C**) There was a trend towards reduced *Bhmt* mRNA in embryonic liver (#*P* = 0.08). (**D**) *Mat1a* and (**E**) *Pemt* mRNA were both significantly decreased due to diet in embryonic liver. Values are means ± SEM of 7–8 embryos/group; *P < 0.05, ***P < 0.005.

At E17.5 the embryo is metabolically active and relies less on maternal metabolism (27). In the FASD group, we observed a decrease in total MTHFR protein levels in embryonic liver (Fig. 7A;*P* < 0.05, unpaired *t*-test), with no differences in isoform ratio (data not shown). These results indicate that the pseudo-MTHFR deficiency is present prenatally. Of additional interest was our finding of decreased *Mthfr* mRNA levels (Fig. 7B;*P* < 0.05, unpaired *t*-test). Changes in *Mthfr* mRNA were not observed due to FASD in the 3-week-old pups or in our earlier study of adult mice (20), suggesting a different regulatory mechanism for MTHFR at the embryonic stage.

We also found a trend towards reduced betaine homocysteine methyltransferase (*Bhmt)* mRNA in FASD embryonic liver (Fig. 7C;*P* = 0.08, unpaired *t*-test). This enzyme utilizes the choline derivative betaine for methionine and SAM synthesis. Key enzymes in the synthesis and utilization of SAM were also decreased by FASD. Expression of methionine adenosyltransferase 1A *(Mat1a)* (Fig. 7D), the enzyme that synthesizes SAM from methionine, and of *Pemt* (Fig. 7E), a major consumer of SAM for phosphatidylcholine synthesis, were reduced (*P* < 0.005, unpaired *t*-test).

**Figure 8:**
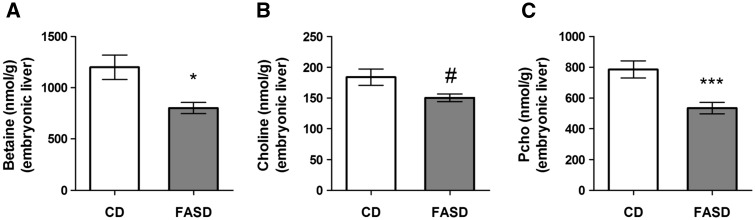
Choline metabolites decreased in E17.5 embryonic liver of FASD mothers. (**A**) Betaine concentration was significantly decreased. (**B**) There was a trend towards reduced choline (#*P* = 0.06) (**C**) Pcho concentration was significantly decreased. Values are means ± SEM of 6 embryos/group; *P < 0.05, ***P < 0.005.

Several of choline/methyl metabolites measured in embryonic liver were not affected by diet (GPC, phosphatidylcholine, sphingomyelin, lysophosphatidylcholine, SAM and SAH) (Supplementary Material, Table S4). However, in FASD liver, betaine was significantly decreased (Fig. 8A;*P* < 0.05, unpaired *t*-test), there was a trend towards decreased choline (Fig. 8B;*P* = 0.06, unpaired *t*-test) and PCho significantly decreased (Fig. 8C;*P* < 0.005, unpaired *t*-test). These observations suggest that the choline/betaine-dependent remethylation pathway is being extensively utilized when folate-dependent remethylation is blocked, as we have previously observed (10,23,28).

**Figure 9:**
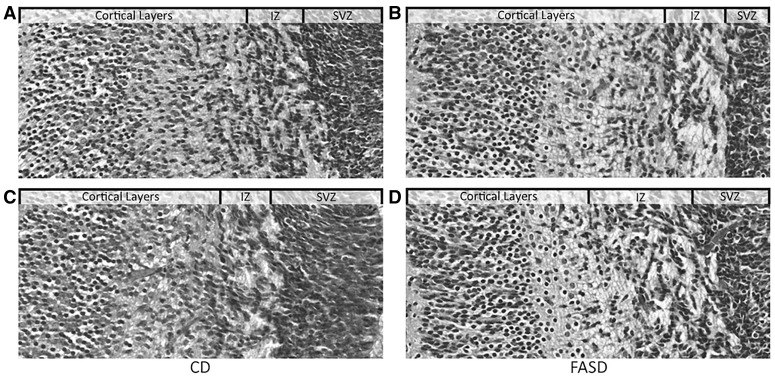
Altered brain development in embryos of FASD mothers. Coronal sections of embryonic brain were stained with H & E. Representative images from embryos in the CD (**A** and **C**) and FASD (**B** and **D**) groups are depicted. Images show the cortical layers, the intermediate zone (IZ) and subventricular zone (SVZ). Cortical layers from FASD embryos appeared to be more organized and developed.

To examine cortical and hippocampal development in E17.5 embryos, we examined H&E stained coronal brain sections. The cortical layers appeared to be more defined in FASD embryos (Fig. 9B and D) compared with CD embryos (Fig. 9A and C), and the cortical cells appeared to be more developed due to their elongated shape. These observations suggest different rates of cortical development due to diet. We also observed increased thickness of the SVZ in FASD embryos (Supplementary Material, Figure S3). Additional experimentation is required to understand the nature of these developmental differences.

### Pregnant mothers at E17.5 showed relatively minor changes in one-carbon metabolism

Total hepatic MTHFR protein was not changed by diet in the pregnant females, although there was an increase in the ratio of phosphorylated to non-phosphorylated MTHFR in the FASD group (*P* < 0.05, unpaired *t*-test, data not shown). Similar to our results in lactating mothers, there was a decrease in *Mtr* mRNA (*P* < 0.05, unpaired *t*-test) due to FASD in livers of pregnant females (Supplementary Material, Fig. S2A).

The placenta, which has significantly higher levels of MTHFR compared with embryonic or maternal liver, did not show differences in MTHFR protein levels due to diet (Supplementary Material, Fig. S2B and C). Consequently, we did not observe any changes in choline metabolites or SAH in placenta or maternal liver. There was a trend for increased SAM in the placenta (*P* = 0.07, unpaired *t*-test); however this small difference may not have biological relevance (Supplementary Material, Table S5). These findings suggest that embryonic folate metabolism may be independent of maternal metabolism at this stage and that FASD has more dramatic consequences on embryos than on dams.

## Discussion

High folate intake is commonly observed in several population sub-groups, including pregnant women, raising concerns about the possible negative consequences on the child’s development (29). We investigated the effect of high folate consumption during pregnancy and lactation on offspring brain function. Three-week-old pups born to FASD mothers had short-term memory impairment. Memory impairment was also observed in our earlier report of *Mthfr^-/-^* mice (10)*.* This similarity is particularly interesting because we found that the pups of FASD mothers had pseudo-MTHFR deficiency. Total MTHFR protein levels were reduced and there was an increased ratio of the phosphorylated (less active) to non-phosphorylated isoform. Our first finding of pseudo-MTHFR deficiency with high folate intake was in the liver of adult male mice maintained on FASD for 6 months (20). The observations in this study of reduced MTHFR protein in livers of pups, embryos and lactating dams suggest that this phenomenon is seen in various ages and sexes, at least for liver. Pregnant dams exhibited increased MTHFR phosphorylation, which would be expected to reduce MTHFR activity (22), but total MTHFR protein was not altered. It is possible that the reduction in total protein requires a longer exposure to the folate-supplemented diet, since we observed both inhibitory mechanisms in lactating mothers. We did not observe changes in MTHFR protein in cortex, hippocampus or placenta due to high folate. Altered expression of liver MTHFR is known to lead to hepatic choline disturbances. Choline is an alternate methyl donor for SAM synthesis, through its metabolite betaine; this alternate pathway is usually upregulated when folate-dependent SAM synthesis is inhibited (20,28). This upregulation may decrease circulating critical choline metabolites and can ultimately affect brain choline utilization for Ach production.

Phosphocholine, the primary storage form of choline, was reduced in liver of 3-week old pups. There was a trend for reduced GPC in pup hippocampus; this decrease could be due to the increased conversion of GPC to choline in order to maintain choline pools (23). Importantly, choline is the precursor of Ach. We found a trend toward decreased ChAT staining in cholinergic neurons in the SI, and a decrease in mRNA levels of *AchE* in cortex. *Chrna7* mRNA levels also tended to decrease in FASD pup cortex; the difference was not significant although *AchE* and *Chrna7* mRNAs were highly positively correlated. *Chrna7* mRNA levels were found to be decreased in post-mortem brains of schizophrenia patients which was likely due to increased expression of the transcription factor Activating Protein-2α (AP-2α), a negative regulator of *AchE* (30). Therefore, it is possible that the observed correlation is due to the co-regulation of these genes by AP-2α. These changes suggest that there is a disturbance in acetylcholine metabolism, which could be contributing to memory impairment in offspring. The hippocampus and the cortex are critical regions for memory and learning (31). The reduced area of the hippocampus in general and the reduced thickness of the dentate gyrus in particular in FASD pups is consistent with the impaired memory and with our earlier work in which we also found decreased hippocampal volume and memory impairment in *Mthfr^-/-^* mice (10).

At E17.5, the hippocampus and upper layers of the cortex are rapidly developing (32) and reduced choline availability may have adverse consequences on embryonic brain development. Maternal choline-deficient diets in mice resulted in decreased proliferation and increased apoptosis in hippocampus (33), and altered neurogenesis and cortical layering, particularly in later born neurons (34). We examined proliferation in hippocampus and cortex at E17.5 using Ki67 (data not shown) but did not observe any differences. More extensive marker analyses at various time points would be useful to understand developmental differences.

Reduced choline availability can also modulate the expression of DNA and histone methyltransferases (35,36). We observed significant disturbances in choline metabolites in FASD embryos, and decreased *Dnmt3a* mRNA in both cortex and hippocampus of FASD pups. *Dnmt3a* plays an important role in brain development and neuronal maturation (37). Mice with a conditional knockout of *Dnmt3a* performed poorly on memory tasks, particularly the NOR (25). Decreased *Dnmt3a* expression would be expected to alter DNA methylation and consequently expression of genes that may be essential for brain development. Increased maternal folic acid intake has been shown to alter methylation and expression of neurodevelopmental genes in a sex-specific manner in newborn pups (24,38). The differences in rate of brain development in this study may be linked to altered *Dnmt3a* expression.

The average weight of embryos per litter was reduced in the FASD group. The observed phenotype is reminiscent of the intrauterine growth restriction observed in low folate conditions (39,40). Therefore, high folate consumption may have similar negative effects to those seen with low folate, particularly since high folate inhibits MTHFR, a critical folate enzyme, and could be inhibiting other folate-dependent enzymes or transporters.

Embryos from FASD mothers had pseudo-MTHFR deficiency with significant disturbances in folate and choline metabolism. The embryo is a system that is rapidly proliferating and developing with high nutrient demands. There were no dramatic changes in metabolites of pregnant mice. These observations suggest that at this stage of development, embryonic folate and choline metabolism is working relatively independently of maternal metabolism.

In contrast to pregnant females, the lactating FASD mice had more marked changes due to diet: reduced MTHFR protein, significant disturbances in one-carbon metabolism and their *Pemt* levels were increased in liver, possibly to maintain choline levels. Maternal metabolism during lactation is critical for maintaining nutrients that are transferred to pups through milk. Nonetheless, we cannot exclude the possibility that the differences between pregnant and lactating mothers are due to the longer period of exposure to FASD and decreased MTHFR protein in lactating dams, instead of a differential response during pregnancy or lactation.

Our findings are in line with recent human studies that examined the effects of excess maternal folate intake on offspring health. Mothers who consumed the highest amounts of folic acid had children with reduced embryonic size (41). The memory impairment in newborn pups from FASD dams in this study is consistent with findings in a Spanish study demonstrating that the children whose mothers consumed over 5mg/day of folic acid periconceptionally had delayed psychomotor development (21). In addition, there is a recent report that high maternal plasma folate was associated with an increased risk of autism spectrum disorder in offspring (42).

In summary, we have shown that high folic acid intake in pregnant mice leads to intrauterine growth delay with altered brain development and impaired memory in offspring. We suggest that these outcomes may be due to pseudo-MTHFR deficiency in both offspring and mothers, with consequent disturbances in choline and methyl metabolism. This study adds to the increasing evidence that over-consumption of folic acid during pregnancy may have negative health consequences and that determining a safe upper limit is critical.

## Materials and Methods

### Animal experimentation and diets

Animal experimentation was conducted according to the guidelines of the Canadian Council on Animal Care and approved by the Animal Care Committee of the Research Institute of the McGill University Health Center. Mice were housed in specific pathogen free facilities with 12 h light/dark cycles with food and water *ad libitium.* At weaning, wild-type C57BL/6 female mice were placed on amino acid defined Control Diet (CD) (2 mg folic acid/kg diet, recommended level for rodents (43) (TD.01369, Harlan Laboratories, Inc., Madison, WI, USA) or Folic Acid Supplemented Diet (FASD) which contained 10 times the amount of folic acid (20 mg folic acid/kg diet, TD.09258, Harlan Laboratories, Inc., Madison, WI, USA). Both diets contained 2.5 g/kg choline bitrate, 3.3 g/kg l-methionine and 1% succinylsulfathiazole, an antibiotic that inhibits folate synthesis by intestinal flora. Five weeks after the start of the diets, females were mated with wild-type C57BL/6 males. The morning of the presence of a vaginal plug was considered as day 0.5 of gestation. For embryo collection, the mice were sacrificed at embryonic day (E) 17.5 of gestation, and maternal and embryonic tissues were collected. In a second group, female mice were maintained on the diets through pregnancy and lactation (until sacrifice). Maternal tissues and tissues from 3-week-old male pups were collected after completion of behavioral testing of pups.

### Behavioral testing

All behavioral tests were performed as described in Ref. (10). Brief descriptions of the methods of individual tests are as follows:

#### Open field test

Mice were placed in the middle of an open field box and allowed to explore for 5 min. The bottom of the open field was divided into 16 equal squares. Recorded videos were analyzed for activity (total number of squares crossed), number of entries into inner squares and outer squares and % time spent in each.

#### Ladder beam test

The apparatus consisted of two Plexiglass walls and metal bars between walls arranged in an irregular pattern, and was placed on top of a mouse housing cage. Recordings were obtained from each mouse and analyzed frame-by-frame. The error score was the combination of total number of errors and the number of steps for each limb.

#### Novel object recognition test

Pups were allowed to habituate to the room for 30 min and then to the apparatus (open field) for 10 min. Animals explored two identical copies of object 1 placed at opposite corners in an open field for 8 min (trial phase), then returned to their cages for one hour. Short-term memory was assessed by allowing animals to explore a familiar object (object 1) and a novel object (object 2) for 5 min (test phase). Both the trial and test phase were recorded and the amount of time exploring objects was assessed from the video recordings by two independent researchers. The discrimination index (DI) was calculated as the ratio of the amount of time animals spent with the novel versus the familiar object. A positive value indicates that the animal preferred to explore the novel object.

#### Y-maze test

Pups were allowed to habituate to the room for 30 min and then placed at the end of the long arm of the maze. Animals were allowed to move through the maze for 8 min. The test was recorded and the series of arm entries were recorded. An alternation is considered as successive entries into the three arms. The number of alternations was recorded and calculated as a percentage of the total arm entries.

### Brain sections and immunohistochemistry

During embryo sacrifice, whole heads were collected and fixed in 4% PFA overnight. They were then paraffin embedded and cut along the coronal axis at 5-μm thickness; mounted sections were 10 sections apart. For the pups, the brain was cut along the sagittal axis and the right hemisphere of the brain was fixed in 4% PFA. Sections were cut at 5-μm thickness. Hematoxylin and eosin (H&E) staining was performed to assess brain morphology. Slides were scanned using the Zeiss Axio Scan.Z1 (Oberkochen, Germany). The examined sections were chosen to be at the same or similar levels between mice. Measurements of area and thickness in the hippocampus were performed using the Zen Lite 2 (Blue Edition) (Zeiss Microscopy, Oberkochen, Germany) by two investigators blinded to groups. The hippocampal area value is an average of four sections per mouse. The thickness values for dentate gyrus are the average of three measurements per section, for four sections per mouse. Cholinergic staining was done as previously described (44) using a ChAT polyclonal goat antibody (Millipore, Billerica, USA). Positive cholinergic neurons were counted in 3–4 sections per animal (5 animals per group) in the Substantia Innominata (SI) at a magnification of 400×. Intensity of staining was measured using ImageJ software.

### Choline metabolites

Choline, betaine, acetylcholine (Ach), glycerophosphocholine (GPC), phosphatidylcholine (PtdCho), sphingomyelin (SM), lysophosphatidylcholine (LPC) and phosphocholine (PCho) were isolated from frozen liver, brain, plasma and placenta using liquid–liquid extraction and SPE purification (45). Stable isotope-dilution liquid chromatography–electrospray ionization tandem mass spectrometry was used for metabolite quantification as previously described (45) with adaptations based on our instrumentation (46). Calibration curves for each metabolite were prepared via serial dilutions of a 100 mM standard stock solution. Ranges were linear between 1 and 100 nmol/extract for choline, betaine, PCho and GPC; 1–100 pmol/extract for Ach; 150–2500 nmol/extract for PtdCho; 6-100 nmol/extract for SM; and 3–50 nmol/extract LPC. Stable isotope-dilution liquid chromatography–electrospray ionization tandem mass spectrometry was also used to measure SAM and SAH concentrations in placenta, liver and brain as previously described (47,48). Calibration curves for SAM and SAH were prepared via serial dilutions of 100 mM stock solutions and ranges were linear between 0.2 and 7.5 nmol/extract for SAM and 0.04–1.2 nmol/extract for SAH. Quality control for all metabolites was monitored using duplicate samples of in-house control materials (i.e. homogenized pooled liver, brain and human placental tissues). The assay imprecision (coefficient of variation) was <5% for betaine; <10% for free choline, SAM, SAH, GPC, PCho, PtdCho, SM, LPC; and <16% for Ach.

### Folates

Quantification of the major folates (folic acid, dihydrofolate, tetrahydrofolate (THF), methenylTHF, methyleneTHF, methylTHF and formylTHF) in mono- and polyglutamated (1–7 glutamates) forms was determined in hippocampus samples by liquid chromatography–electrospray ionization tandem mass spectrometry, as previously described (49). The relative abundance of individual folate forms as a proportion of total folate was compared between groups.

### Quantitative real-time PCR

RNA was extracted from frozen liver (∼15 mg) using the RNeasy Mini Kit (Qiagen, Toronto, Canada). RNA from brain was extracted using the AllPrep DNA, RNA and miRNA Universal kit (Qiagen, Toronto, Canada). cDNA synthesis was done using the iScript cDNA Synthesis kit (BioRad, Quebec, Canada) and quantitative real-time PCR reactions were done using the SSoAdvanced Universal SYBR Green Supermix. For all tissues, the following reference genes were tested: glyceraldehyde-3-phosphate dehydrogenase (*Gapdh), β-*2-microglobulin *(B2m), β –actin (Actb)* and tyrosine 3-monooxygenase/tryptophan 5-mooxygenase activation protein, zeta polypeptide (*Ywhaz).* The normalization factor was calculated for each tissue using the two most stably expressed genes by geNorm v.3.4 (Ghent University Hospital Center for Medical Genetics) (50). Primers were designed using the Primer-BLAST (National Center for Biotechnology Information) (51). A complete list of primer sequences and reactions conditions is included in Supplementary Material, Table S6.

### Western blotting

Protein extracts were prepared as before (20) and the concentration was determined by Bradford Assay with a bovine serum albumin standard. Western blots were performed as previously described (20). Primary antibodies were against MTHFR (2), ChAT (Millipore, Billerica, USA), GAPDH (Cell Signaling Technology, Boston, USA) and *β-*actin (Sigma-Aldrich). Secondary antibodies were horseradish peroxidase (HRP)-conjugated donkey anti-rabbit IgG (GE Healthcare, Mississauga, Canada) and HRP-conjugated donkey anti-goat IgG (Santa Cruz Biotechnology, Santa Cruz, USA), as appropriate. Detection was achieved using ECL Prime Western Blotting Detection Reagent (GE Healthcare, Mississauga, Canada). Bands were quantified by densitometry using Quantity One *4.1.0* (Bio-Rad) and normalized to loading control (GAPDH or *β-*actin).

### Statistical analysis

Statistical analysis was performed with Graph Pad software package 5.01 (GraphPad Software, La Jolla, USA, 1995). All data are presented as mean ± standard error of the mean (SEM). Statistical outliers, determined by using Grubb’s test (GraphPad Software), were removed from the analysis. Data were analyzed using an unpaired *t*-test. If the variance was statistically significant, Welsh’s correction was applied. Results were considered statistically significant at *P* ≤ 0.05.

## Supplementary Material

Supplementary Material is available at *HMG* online.

## Supplementary Material

Supplementary Data
