# Supplementary material for: High dietary folate in pregnant mice leads to pseudo-MTHFR deficiency and altered methyl metabolism, with embryonic growth delay and short-term memory impairment in offspring
Source: Hum Mol Genet. 2017 Jan 6;26(5):888–900. doi: 10.1093/hmg/ddx004 (PMC5409086; doi:10.1093/hmg/ddx004)
Supplement: Supplementary Data [file ddx004_Supp.docx]

**Supplementary Table 1.**

Maternal high folate intake did not alter the distribution of folate derivatives in hippocampus of 3-week-old pups

| Folate Derivative  (% total folate) | CD | FASD |
| --- | --- | --- |
| DHF ^*^ | 3.88 ± 0.24 | 2.80 ± 0.37 |
| THF | 41.48 ± 1.91 | 41.09 ± 2.80 |
| Methenyl-THF | 1.18 ± 0.15 | 1.56 ± 0.33 |
| Methylene-THF | 0.79 ± 0.18 | 0.99 ± 0.05 |
| 5-Methyl-THF | 49.4 ± 1.39 | 48.56 ± 3.16 |
| Formyl-THF | 3.24 ± 0.50 | 4.03 ± 0.65 |

n=3/group

Values are means ± SEM (analyzed by t-test)

^*^p =0.03

**Supplementary Table 2.**

Maternal high folate intake altered the concentration of some choline metabolites in liver or hippocampus of 3-week-old pups

| Metabolite (nmol/g) | Liver | | | Hippocampus | | |
| --- | --- | --- | --- | --- | --- | --- |
|  | CD | | FASD | CD | FASD | |
| Choline | 187.6 ± 22.5 | | 162.2 ± 22.6 | 106.7 ± 9.9 | 90.5 ± 7.4 | |
| Betaine | 428 ± 57.1 | | 387 ± 34.8 | 21 ± 1.5 | 21 ± 2.3 | |
| GPC ^#^ | 1121 ± 109 | | 1147 ± 95 | 466 ± 37 | | 372 ± 26 |
| Phosphatidylcholine | 23469 ± 497 | | 22971 ± 80 | 32070 ± 549 | 30453 ± 1901 | |
| Phosphocholine * | 234.8 ± 22.7 | 164.1 ±10.4 | | 674.9 ± 35.2 | 650.5 ± 32.4 | |
| Sphingomyelin | 2109 ± 53 | | 2010 ± 97 | 1380 ± 34 | 1313 ± 84 | |
| Lysophosphatidylcholine | 283.6 ± 10.6 | | 280.3 ± 16.4 | n/a | n/a | |
| Acetylcholine | n/a | | n/a | 2.9 ± 0.6 | 2.4 ± 0.3 | |
| SAM | 72.3 ± 6.4 | | 69.2 ± 3.1 | n/a | n/a | |
| SAH | 23.4 ± 1.2 | | 22.7 ± 1.0 | n/a | n/a | |

n=6-7/group

Values are means ± SEM (analyzed by t-test)

^#^p=0.06 (in hippocampus); *p<0.05

n/a, not available

**Supplementary Table 3.**

High folate intake did not significantly affect concentration of choline metabolites in liver or plasma of lactating mothers

| Metabolite | Liver  (nmol/g) | | Plasma  (nmol/ml) | |
| --- | --- | --- | --- | --- |
|  | CD | FASD | CD | FASD |
| Choline | 102.2 ± 22.0 | 113.5 ± 21.9 | 11.9 ± 1.6 | 11.2 ± 1.2 |
| Betaine | 172 ± 46.1 | 169 ± 31.7 | 36 ± 7.9 | 40 ± 5.9 |
| GPC ^#^ | 524 ± 44 | 490 ± 64 | 35 ± 5 | 25 ± 2 |
| Phosphatidylcholine | 22431±1142 | 23447 ± 1030 | 1708 ± 182 | 1954 ± 112 |
| Phosphocholine | 210.8 ± 30.5 | 281.0 ± 34.5 | n/a | n/a |
| Sphingomyelin | 527.6 ± 50 | 566.5 ± 30 | 37.9 ± 6 | 59.0 ± 12 |
| Lysophosphatidylcholine | 315.2 ± 23.3 | 320.6 ±14.1 | 268.8 ± 20.2 | 305.4 ± 15.9 |

n=6-7/group

Values are means ± SEM (analyzed by t-test)

^#^p=0.06 (in plasma)

n/a, not available

**Supplementary Table 4.**

Maternal high folate intake significantly changed the concentration of choline, betaine and phosphocholine in embryonic liver

| Metabolite (nmol/g) | CD | FASD |
| --- | --- | --- |
| Choline ^#^ | 180 ± 13.3 | 150.6 ± 6.2 |
| Betaine^*^ | 1199 ± 118.4 | 803 ± 54.2 |
| GPC | 691.9 ± 75 | 541.9 ± 38 |
| Phosphatidylcholine | 14771 ± 472 | 14660 ± 196 |
| Phosphocholine ^***^ | 785.2 ± 55.9 | 534.1 ± 38.1 |
| Sphingomyelin | 2109 ± 119 | 2204 ± 33 |
| Lysophosphatidylcholine | 180.0 ± 8.8 | 166.0 ± 4.2 |
| SAM | 119.1 ± 4.5 | 118.9 ± 5.1 |
| SAH | 4.5 ± 0.3 | 4.1 ± 0.2 |

n=7/group

Values are means ± SEM (analyzed by t-test)

^#^p=0.06 ; *p<0.05; ***p<0.005

**Supplementary Table 5.**

High folate intake did not significantly change the concentration of choline metabolites, SAM or SAH in E17.5 placenta^1^ or maternal liver ^2^

| Metabolite (nmol/g) | Placenta | | Liver | | |
| --- | --- | --- | --- | --- | --- |
|  | CD | FASD | CD | | FASD |
| Choline | 655.2 ± 56.5 | 725.4 ± 89.1 | 58.3 ± 10.2 | 49.4 ± 5.9 | |
| Betaine | 6937 ± 773.4 | 7485 ± 654.3 | 256 ± 20.3 | 228 ± 29.0 | |
| GPC | 390 ± 39 | 454 ± 22 | 239 ± 21 | 244 ± 15 | |
| Phosphatidylcholine | 16954 ± 315 | 17407 ± 460 | 15718 ± 907 | 15290 ± 660 | |
| Phosphocholine | 449.3 ± 26.5 | 470.7 ± 18.4 | 77.1 ±14.5 | 76.5 ± 8.0 | |
| Sphingomyelin | 4479 ± 339 | 4640 ± 262 | 2040 ± 119 | 2080 ± 18 | |
| Lysophosphatidylcholine | n/a | n/a | 340.8 ± 16.4 | 374.1± 4.1 | |
| SAM ^#^ | 37.7 ± 1.2 | 41.4 ± 1.4 | 68.8 ± 7.6 | 68.5 ± 7.4 | |
| SAH | 1.38 ± 0.13 | 1.37 ± 0.14 | 26.7 ± 0.6 | 28.0 ± 0.6 | |

^1^ n=7/group ; ^2^ n=5/group

Values are means ± SEM (analyzed by t-test)

^#^ p=0.07

n/a, not available

**Supplementary Table 6. Primers for quantitative real-time PCR**

| Gene | Direction | Primer Sequence | Amplicon Size | Tm (C) | Reference |
| --- | --- | --- | --- | --- | --- |
| *Gapdh* | Forward | CAGGAGCGAGACCCCACTAACAT | 74 | 62 | 20 |
|  | Reverse | AAGACACCAGTAGACTCCACGAC |  |  |  |
| *Actin* | Forward | CTGACGGCCAGGTCATCACTA | 105 | 60 | 20 |
|  | Reverse | TAGTTTCATGGATGCCACAGGAT |  |  |  |
| *Ywhaz* | Forward | TGCTGGTGATGACAAGAAAGGA | 119 | 60 | 20 |
|  | Reverse | TGAGGGCCAGACCCAGTCT |  |  |  |
| *B2m* | Forward | ATGCTATCCAGAAAACCCCTCAA | 100 | 60 | 20 |
|  | Reverse | GCGGGTGGAACTGTGTTACG |  |  |  |
| *Mthfr* | Forward | GATGAGGCGCAGAATGGACT | 104 | 62 | 20 |
|  | Reverse | TCCGGTCAAACCTGGAGATG |  |  |  |
| *Mtr* | Forward | ACTCATGGCACAGGAGGGAAGAAA | 94 | 60 | 20 |
|  | Reverse | TGCCCTTCACAAGAGCATACTCCA |  |  |  |
| *Chdh* | Forward | TGGCTGAAACTGAGAAGTGCCAAC | 115 | 60 | 20 |
|  | Reverse | TTTCTCGTGACAGCCTCACACACT |  |  |  |
| *Bhmt* | Forward | TCCACCCATCTAGGAGACCAATCT | 91 | 60 | 20 |
|  | Reverse | ACACGCCCACCAGCCTTTGAATTA |  |  |  |
| *Pemt* | Forward | GAATGTGGTAGCGAGATGGGA | 119 | 62 | 20 |
|  | Reverse | GGGAGCGGAGGATGTTCAAA |  |  |  |
| *AchE* | Forward | GTTTGAGGGAGCTGTGTTGGTA | 120 | 60 | 45 |
|  | Reverse | AGCCGTTGATCCAGCAGACCT A |  |  |  |
| *Chrna7* | Forward | TTCCGTGCCCTTGATAGCACA | 70 | 60 | 45 |
|  | Reverse | TGTCACGACCACTGAGAGGC |  |  |  |
